# Effect of etanercept therapy on psoriasis symptoms in patients from Latin America, Central Europe, and Asia: a subset analysis of the PRISTINE trial

**DOI:** 10.1186/s12895-015-0028-8

**Published:** 2015-05-21

**Authors:** L. Kemeny, M. Amaya, P. Cetkovska, N. Rajatanavin, W-R. Lee, A. Szumski, L. Marshall, E. Y. Mahgoub, E. Aldinç

**Affiliations:** Department of Dermatology and Allergology, University of Szeged, Szeged, Hungary; Hospital San Lucas, Monterrey, Nuevo Leon Mexico; Department of Dermatovenereology, Charles University Hospital, Pilsen, Czech Republic; Division of Dermatology, Ramathibodi Hospital, Mahidol University, Bangkok, Thailand; Department of Dermatology, Shuang Ho Hospital, Taipei, Taiwan; Global Innovative Pharma, Pfizer, Collegeville, PA USA; Global Innovative Pharma, Pfizer, New York, NY USA

**Keywords:** Etanercept, Psoriasis treatment, Asia, Central Europe, Latin America

## Abstract

**Background:**

Psoriasis prevalence and characteristics in Asia, Central Europe, and Latin America have not been thoroughly investigated and there are no large trials for biologic treatments for patients from these regions. The goal of this analysis was to report clinical response to anti-tumor necrosis factor-alpha treatment in these patients.

**Methods:**

Patients from Argentina, Czech Republic, Hungary, Mexico, Taiwan, and Thailand (*N* = 171) were included in this subset analysis of the PRISTINE trial. Patients with stable moderate-to-severe plaque psoriasis were blinded and randomized to receive etanercept 50 mg once weekly (QW) or biweekly (BIW) for 12 weeks, followed by 12 weeks of open-label QW treatment with etanercept 50 mg through week 24 (QW/QW vs. BIW/QW). Concomitant methotrexate (≤20 mg/week) and mild topical corticosteroids or other agents were permitted at the physician’s discretion, in accordance with therapeutic practice.

**Results:**

As early as week 8, 26.7 % in the etanercept QW group and 44.0 % in the BIW group achieved Psoriasis Area and Severity Index (PASI) 75. At weeks 12 and 24, respectively, PASI 75 increased to 39.5 % and 62.8 % in the QW/QW group and 66.7 % and 83.3 % in the BIW/QW group. PASI 75 was significantly different between treatment groups from week 8 through the end of study (*p* < 0.05). The Kaplan-Meier estimate of the proportions achieving PASI 75 in QW/QW and BIW/QW groups, respectively, was 27.4 % and 45.8 % through week 8; 41.9 % and 68.7 % through week 12; and 72.5 % and 95.2 % through week 24.

**Conclusions:**

Treatment with etanercept 50 mg provided rapid relief of psoriasis symptoms in patients from Asia, Central Europe, and Latin America. A more rapid response was observed in patients who received BIW treatment for the first 12 weeks which was sustained after reducing to QW dosing for the subsequent 12 weeks. Response rates were similar to those observed in the overall PRISTINE population.

**Trial registration:**

ClinicalTrials.gov identifier NCT00663052.

## Background

Psoriasis is a chronic inflammatory skin condition characterized by exacerbations and remissions and estimated to affect approximately 125 million people (2–3 %) worldwide [1]. In the United States, where such data are available, the prevalence of psoriasis varies among ethnicity, with 0.47 % of Chinese [2], 1.3 % of African Americans and 1.6 % of Hispanic affected compared with 3.6 % of Caucasians [1]. As such, it is possible that patients from different parts of the world may respond differently to treatment.

The goal of treatment in psoriasis is to alleviate symptoms as rapidly as possible and maintain the response over time. Current treatment guidelines in both the United States and Europe support the combination of topical and systemic therapies, including biologic agents, in order to achieve these goals [3–6]. Although the effectiveness of biologic agents is well-established through clinical trials in the United States and Europe [5–7], these agents have not been studied extensively in many parts of the world.

The PRISTINE trial was a multinational, randomized, double-blind study in patients with moderate-to-severe plaque psoriasis in which investigators evaluated the efficacy and safety of two dosing regimens of etanercept [8]. This trial included patients from Argentina, Czech Republic, Hungary, Mexico, Taiwan, and Thailand. The objective of the subset analysis reported here was to evaluate the efficacy of etanercept therapy in patients from countries in Asia, Central Europe, and Latin America.

## Methods

### Study details

**Fig. 1:**
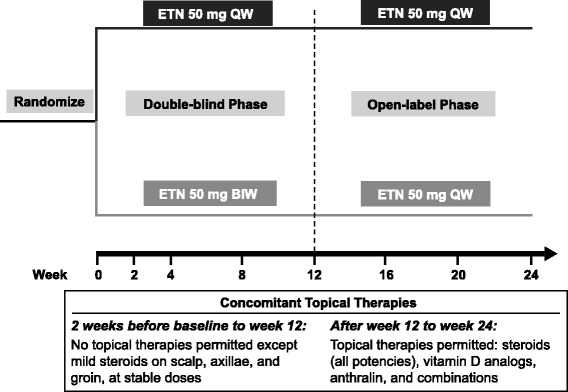
PRISTINE study design. BIW: twice weekly; QW: once weekly

The details of the PRISTINE trial have been previously published [8]. Briefly, patients ≥18 years of age with stable moderate-to-severe plaque psoriasis were randomized to receive 50 mg etanercept subcutaneously either once weekly (QW) or twice weekly (BIW) for 12 weeks, after which all patients received open-label, 50 mg etanercept subcutaneously QW for an additional 12 weeks, i.e. QW/QW or QW/BIW dosing groups (Fig. 1). Concomitant methotrexate was allowed (≤20 mg/week) if doses were stable from at least 28 days prior to baseline through the end of study. Only mild topical corticosteroids were permitted on scalp, axillae and groin for first 12 weeks; topical medications (corticosteroids of all potencies, vitamin D analogues and combination products) were allowed as needed, at physician’s discretion, during the second 12 weeks, consistent with therapeutic practice. Of the 273 patients enrolled in the PRISTINE trial, 171 patients were eligible for this subset analysis.

The study protocol was reviewed and approved by an independent Ethics Committee prior to initiation. The study was conducted in compliance with the ethical principles of the Declaration of Helsinki and the International Conference on Harmonization Good Clinical Practice Guidelines. The PRISTINE trial is registered on ClinicalTrials.gov, identifier NCT00663052.

### Study endpoints

Primary efficacy was measured as the proportion of patients achieving 50 %, 75 %, or 90 % improvement in Psoriasais Area and Severity Index (PASI), PASI 50, PASI 75, and PASI 90, respectively, at weeks 8, 12, and 24. Other efficacy endpoints included the percentage of patients who achieved a status of “clear” or “almost clear” on the Physician’s Global Assessment (PGA) of psoriasis, time to achieving PGA first “clear” or “almost clear” status, and percentage reduction in affected body surface area (BSA). Health-related quality of life (HRQoL) measures included Dermatology Life Quality Index (DLQI) [9], EuroQoL-5 Dimension (EQ-5D™) [10, 11], Work Productivity and Activity Impairment scale (WPAI) [12] and Functional Activity in Chronic Therapy (FACIT) [13].

### Statistical analyses

For continuous efficacy parameters, treatment groups were compared in 1-way analysis of variance for baseline parameters or in analysis of covariance models of week 12/24 change from baseline parameters with treatment group as a factor and baseline measurement as a covariate. For dichotomous or categorical parameters, Fisher’s exact test was used. The last observation was carried forward for patients for whom data were not available at any time point.

## Results

### Patients

**Fig. 2:**
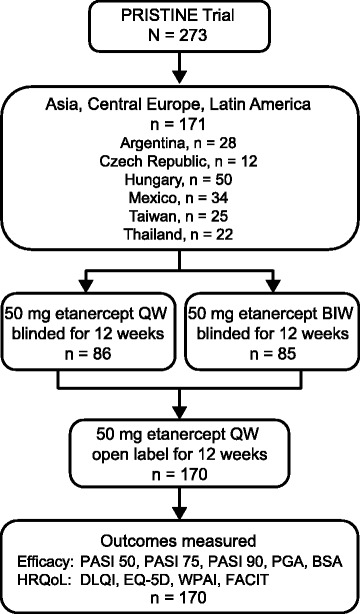
Subset analysis design**.** BIW: twice weekly; BSA: body surface area; DLQI: Dermatology Life Quality Index; EQ-5D: EuroQoL-5 Dimension; FACIT: Functional Activity in Chronic Therapy; HRQoL: health-related quality of life; PASI: Psoriasis Area and Severity Index; PGA: Physician Global Assessment of psoriasis; QW: once weekly; WPAI: Work Productivity and Activity Impairment scale

Of the 273 patients enrolled in the PRISTINE trial, all 171 patients from Asia (Taiwan, *n* = 25; Thailand, *n* = 22), Central Europe (Czech Republic, *n* = 12; Hungary, *n* = 50), and Latin America (Argentina, *n* = 28; Mexico, *n* = 34) were included in the analysis. Since the number of patients from each region was small, they were pooled together for this subset analysis (Fig. 2).

The baseline demographics were similar between the etanercept 50 mg QW/QW and BIW/QW treatment groups (Table 1). In addition, the history of psoriasis, the extent of the disease, the presence and duration of psoriatic arthritis, and baseline HRQoL measures were also similar between the two groups of patients (Table 1). The baseline characteristics of the patients in this subset were comparable to those from the overall PRISTINE population (Table 2).

**Table 1:** Baseline characteristics of all randomized patients from Asia, Central Europe, and Latin America (*n* = 171^a^)

Characteristic	QW/QW (*n* = 86)	BIW/QW (*n* = 85)	*p* value
Age, years	45.8 (13.0)	44.8 (12.0)	0.581
Male gender, n ( %)	64 (74.4)	63 (74.1)	1.000
Race, n (%)			0.898
White	44 (51.2)	46 (54.1)	
Asian	25 (29.1)	22 (25.9)	
Other	17 (19.8)	17 (20.0)	
Body weight, kg	87.3 (19.3)	84.5 (17.1)	0.311
Body mass index, kg/m^2^			
Male	28.9 (5.1)	28.2 (4.4)	0.438
Female	32.8 (8.4)	30.4 (6.7)	0.295
Waist to hip ratio			
Male	1.0 (0.1)	1.0 (0.1)	0.289
Female	0.9 (0.1)	0.9 (0.1)	0.501
Current smokers, n (%)	29 (33.7)	25 (29.4)	0.622
Psoriasis disease duration, years	17.0 (10.8)	15.8 (7.8)	0.440
PASI total score	22.2 (9.7)	22.4 (9.4)	0.905
PGA score	3.4 (0.8)	3.4 (0.7)	0.955
Affected body surface area, %	38.0 (22.6)	36.9 (19.6)	0.743
History of psoriatic arthritis, n (%)	32 (37.2)	32 (37.7)	1.000
Duration of psoriatic arthritis, years	7.5 (7.1)	7.7 (5.6)	0.903
Secondary diagnosis of diabetes, n (%)	9 (10.5)	12 (14.1)	0.494
Secondary diagnosis of hypertension, n (%)	37 (43.0)	37 (43.5)	1.000
DLQI score	14.8 (8.4)	14.8 (7.2)	0.967
EQ-5D score	0.6 (0.3)	0.6 (0.3)	0.856
WPAI: % activity impairment due to problem	39.3 (31.2)	42.6 (30.5)	0.487
WPAI: % impairment while working due to problem	23.2 (25.0)	25.8 (24.8)	0.603
WPAI: % overall work impairment due to problem	27.1 (26.0)	27.1 (24.8)	0.999
WPAI: % work time missed due to problem	8.3 (20.7)	4.8 (19.5)	0.378
FACIT score	35.9 (11.4)	37.3 (9.4)	0.368

^a^Data are given as mean (SD) unless otherwise specified

*BIW* twice weekly, *DLQI* Dermatology Life Quality Index, *EQ-5D* EuroQOL 5 Dimension, *FACIT* Functional Activity in Chronic Therapy, *PASI* Psoriasis Area and Severity Index, *PGA* Physician Global Assessment of psoriasis, *QW* once weekly, *WPAI* Work Productivity and Activity Impairment scale

**Table 2:** Baseline characteristics of patients from Asia, Central Europe, and Latin America compared with total PRISTINE population^a^

Characteristic	Patients from Asia, Central Europe, and Latin America (*n* = 171)	Full PRISTINE population (*n* = 273)
Age, years	45.3 (12.5)	43.9 (12.7)
Male gender, n (%)	127 (74.3)	190 (69.6)
Ethnicity, n (%)		
White	90 (52.6)	174 (63.7)
Asian	47 (27.5)	64 (23.4)
Other	34 (19.9)	35 (12.8)
Body weight, kg	85.9 (18.3)	85.1 (18.6)
Body mass index, kg/m^2^		
Male	28.5 (4.8)	28.3 (4.6)
Female	31.6 (7.6)	29.6 (7.5)
Waist to hip ratio		
Male	1.0 (0.1)	1.0 (0.1)
Female	0.9 (0.1)	0.9 (0.1)
Prior smokers, n (%)	57 (33.7)	109 (40.2)
Psoriasis disease duration, years	16.4 (9.4)	17.3 (10.6)
PASI total score	22.3 (9.6)	21.2 (9.4)
PGA score	3.4 (0.8)	3.4 (0.8)
Affected body surface area, %	37.4 (21.1)	33.0 (20.2)
History of psoriatic arthritis, n (%)	64 (37.4)	84 (30.8)
Duration of psoriatic arthritis, years	7.6 (6.3)	8.2 (8.0)
Secondary diagnosis of diabetes, n (%)	21 (12.3)	26 (9.5)
Secondary diagnosis of hypertension, n (%)	74 (43.3)	97 (35.5)

^a^Data are given as mean (standard deviation) unless otherwise specified

*PASI* Psoriasis Area and Severity Index, *PGA* Physician Global Assessment of psoriasis

### Efficacy analyses

**Fig. 3:**
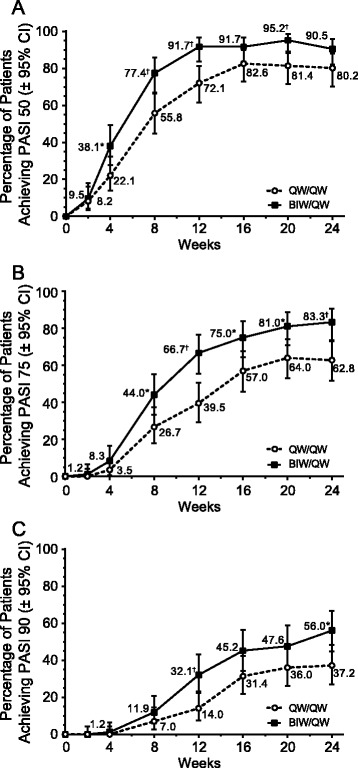
Percentage of PASI 50 **a**, 75 **b**, and 90 **c** responders by treatment group (LOCF data). * *p* < 0.05; ^†^ *p* < 0.01. BIW: twice weekly; LOCF: last observation carried forward; PASI: Psoriasis Area and Severity Index; QW: once weekly

**Fig. 4:**
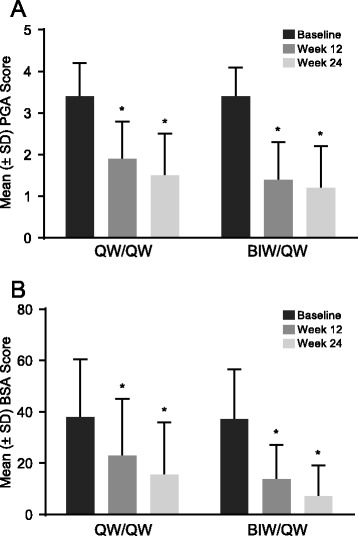
Effect on PGA **a** and BSA **b** scores in response to etanercept by treatment group (LOCF data). * *p* < 0.0001. BIW: twice weekly; BSA: body surface area; LOCF: last observation carried forward; PGA: Physician’s Global Assessment; QW: once weekly

There were more patients achieving PASI 50, PASI 75, and PASI 90 in the group that received etanercept 50 mg BIW than in the group that received etanercept 50 mg QW over the time course of the study (Fig. 3). Statistically significant difference between the two treatment groups was evident as early as week 4 in PASI 50. Statistically significant difference in PASI 75 was observed by week 8 and in PASI 90 by week 12 (Fig. 3). After 12 weeks of treatment, i.e. at the end of the blinded phase of the study, 72 %, 40 % and 14 % of patients in the QW/QW group and 92 %, 67 % and 32 % in the BIW/QW group achieved PASI 50, PASI 75 and PASI 90, respectively. Kaplan-Meier estimates of the proportions of patients achieving first PASI 50, PASI 75 and PASI 90 responses by weeks 8, 12 and 24 also indicate a strong beneficial response in both treatment groups (Table 3). Improvements from baseline were also observed in PGA and BSA scores (*p* < 0.0001) at weeks 12 and 24 in both treatment groups (Fig. 4). By week 12, 36 % and 56 % of patients in the QW/QW and BIW/QW groups, respectively, exhibited a PGA status of clear or almost clear (Table 4). By week 24, the number of patients with clear or almost clear status increased to 57 % and 71 % in the QW/QW and BIW/QW groups, respectively. For the achievement of first PGA of clear/almost clear response, there was a statistically significant difference between the time-to-event curves for the QW/QW and BIW/QW treatment arms (*p* = 0.0112) and a significantly higher median time-to-response for the QW/QW group (113 days; 95 % confidence interval [CI]: 85–141) compared with the BIW/QW group (85 days; 95 % CI: 59–86). The efficacy parameters are summarized in Table 4.

**Table 3:** Kaplan-Meier rate estimates: proportions of patients achieving first PASI 50, 75, and 90, by treatment group

PASI Response	% of Patients (95 % CI)					
	Week 0–8		Week 0–12		Week 0–24	
	QW/QW *n* = 86	BIW/QW *n* = 84	QW/QW *n* = 86	BIW/QW *n* = 84	QW/QW *n* = 86	BIW/QW *n* = 84
PASI 50	59.5\ (49.3, 70.0)	79.5 (70.3, 87.4)	75.5 (65.8, 84.1)	92.8 (85.9, 97.0)	90.5 (82.8, 95.6)	98.8 (94.2, 99.9)
PASI 75	27.4 (19.1, 38.3)	45.8 (35.8, 57.1)	41.9 (32.2, 53.2)	68.7 (58.6, 78.3)	72.5 (62.5, 81.6)	95.2 (89.1, 98.4)
PASI 90	7.2 (3.3, 15.2)	13.3 (7.6, 22.7)	15.6 (9.4, 25.3)	32.5 (23.6, 43.7)	47.7 (37.5, 59.1)	61.5 (51.2, 71.8)

*BIW* twice weekly, *CI* confidence interval, *PASI* Psoriasis Area and Severity Index, *QW* once weekly

**Table 4:** Summary of improvements in efficacy measures in response to etanercept by treatment group

Response/Parameter	Week 12		Week 24	
	QW/QW *n* = 86	BIW/QW *n* = 84	QW/QW *n* = 86	BIW/QW *n* = 84
PASI 50, % of patients (95 % CI)	72.1 (61.4, 81.2)	91.7* (83.6, 96.6)	80.2 (70.2, 88.0)	90.5 (82.1, 95.8)
PASI 75, % of patients (95 % CI)	39.5 (29.2, 50.7)	66.7* (55.5, 76.6)	62.8 (51.7, 73.0)	83.3* (73.6, 90.6)
PASI 90, % of patients (95 % CI)	14.0 (7.4, 23.1)	32.1* (22.4, 43.2)	37.2 (27.0, 48.3)	56.0* (44.7, 66.8)
Adjusted mean change from baseline in total PGA score, mean (SEM)	−1.5^†^ (0.1)	−2.0*^†^ (0.1)	−1.9^†^ (0.1)	−2.2*^†^ (0.1)
PGA clear/almost clear, % of patients (95 % CI)	36.0^†^ (26.0, 47.1)	56.0*^†^ (44.7, 66.8)	57.0^†^ (45.8, 67.6)	71.4^†^ (60.5, 80.8)
Adjusted mean % change in affected body surface area, mean (SEM)	−14.6^†^ (1.7)	−23.2*^†^ (1.7)	−21.7^†^ (1.5)	29.6*^†^ (1.5)

Missing data were imputed using the last observation carried forward method

*p < 0.05 between treatment groups at the same time point. ^†^ *p* < 0.01 from baseline within treatment group

*BIW* twice weekly, *PASI* Psoriasis Area and Severity Index, *PGA* Physician’s Global Assessment, *QW* once weekly, *SEM* standard error of the mean

### HRQoL analyses

Statistically significant (*p* < 0.001) improvements from baseline in all measures of HRQoL were observed in both treatment groups by week 12 and were maintained to week 24 (Table 5). In addition, the difference in the observed improvement in DLQI and EQ-5D scores at week 12 between the two treatment groups was statistically significant (*p* < 0.05).

**Table 5:** Summary of improvements in HRQoL measures in response to etanercept by treatment group

Adjusted mean change from baseline	Week 12		Week 24	
	QW/QW *n* = 86	BIW/QW *n* = 84	QW/QW *n* = 86	BIW/QW *n* = 84
DLQI, mean (SEM)	−8.4^†^ (0.5)	−10.8^†^ (0.5)	−9.5^†^ (0.6)	−11.0^†^ (0.6)
EQ-5D, mean (SEM)	0.2 (0.0)	0.3* (0.0)	0.2* (0.0)	0.3* (0.0)
WPAI: % activity impairment due to problem, mean (SEM)	−19.1^†^ (2.3)	−25.1^†^ (2.3)	−22.1^†^ (2.2)	−27.3^†^ (2.3)
WPAI: % impairment while working due to problem, mean (SEM)	−7.5 (2.5)^†^	−16.3 (2.6)*^†^	−11.8 (2.0)^†^	−18.8 (2.0)*^†^
WPAI: % overall work impairment due to problem, mean (SEM)	−7.8 (3.3)^†^	−15.1 (3.2)^†^	−13.5 (2.8)^†^	−16.3 (2.8)^†^
WPAI: % work time missed due to problem, mean (SEM)	−3.4 (2.6)^†^	0.4 (2.5)^†^	−6.1 (2.3)^†^	0.6 (2.3)*^†^
Total FACIT, mean (SEM)	3.2^†^ (0.7)	4.5^†^ (0.7)	4.5^†^ (0.8)	4.3^†^ (0.8)

Missing data were imputed using the last observation carried forward method

*p < 0.05 between treatment groups at the same time point. ^†^ *p* < 0.01 from baseline within treatment group

*BIW* twice weekly, *DLQI* Dermatology Life Quality Index, *EQ-5D* EuroQoL 5 Dimension, *FACIT* Functional Activity in Chronic Therapy, *HRQoL* health-related quality of life, *QW* once weekly, *SEM* standard error of the mean, *WPAI* Work Productivity and Activity Impairment scale

### Safety analyses

Individual safety analysis by country or region was not performed since the trial was designed to randomize all enrolled patients and not stratified by geographic location. The complete safety data for the PRISTINE trial have been reported before [8]. Briefly, etanercept was well tolerated. The most commonly reported (≥5 % of patients) treatment-emergent adverse events were nasopharyngitis, headache, elevated blood insulin, diarrhea, injection-site erythema, pharyngitis, arthralgia, fatigue and injection-site reaction. Seven patients of 273 (2.6 %) reported serious adverse events and nine patients discontinued treatment due to an adverse event. There was no incidence of tuberculosis, opportunistic infections, or deaths reported.

## Discussion

Guidelines for the treatment of psoriasis have been well established in the United States and Western Europe [3–6] and, more recently, in the Czech Republic [14] and Mexico [15]. These same treatment paradigms have been used in other parts of the world with the expectation that there would be similar responses. However, there have been few, if any, formal evaluations of responses to any specific treatment in patients from other parts of the world. The fact that the prevalence of psoriasis in Hispanic, African Americans, and other ethnic groups is less than half of that observed in Caucasians (1.4 %–1.6 % vs. 3.6 %, respectively) [1] suggests that it may be important to at least review and re-evaluate the responses of patients from other ethnic backgrounds and countries.

In this post-hoc, subset analysis, we examine the responses of patients from six countries (Argentina, Czech Republic, Hungary, Mexico, Taiwan, and Thailand) in three regions of the world (Asia, Central Europe, and Latin America) in which there are no current guidelines for the treatment of psoriasis other than in the Czech Republic [14] and Mexico [15]. Of the 273 patients originally enrolled in the PRISTINE trial, 171 patients were from these three regions. However, since the number of patients from each of the six countries was small, they were pooled for descriptive statistical analyses.

The percentages of patients achieving PASI 50, PASI 75 or PASI 90 in response to etanercept treatment were numerically greater in this subset than the corresponding percentages in the overall PRISTINE population [8] at both the 12- and 24-week time points. Similarly, the percentage of patients achieving a PGA status of clear or almost clear in response to etanercept treatment was also numerically greater in this subset than in the overall PRISTINE study population. Even though some outcomes appear to have slightly better responses numerically for this subpopulation compared with the overall study population [8], the underlying cause for these differences is unclear. This could be related to shorter psoriatic arthritis disease duration; slightly higher disease severity, e.g. BSA and PASI, at baseline for this subpopulation, allowing for greater improvement; slightly higher body mass index and smaller waist-to-hip ratio among the females in this subpopulation; slightly fewer Caucasians; slightly higher number of patients with secondary diagnosis of psoriatic arthritis, diabetes or hypertension; or random chance. Since the study was designed to randomize all enrolled patients without stratification by their geographic location, the patients from these six countries were not homogenously distributed between the two treatment groups. Thus, any analysis comparing the responses of the subpopulation from these six countries with those from the rest of the enrolled patients could introduce bias in the results which could be random or due to regional differences, e.g., accepted standard of care.

The Kaplan-Meier estimates for time to first response also demonstrate the rapidity with which patients in this subset experienced the benefits of etanercept treatment. As might be expected, the response time was shorter for those receiving etanercept BIW (median time 85 days, 95 % CI: 59–86 days) compared with those receiving etanercept QW during the first 12 weeks of the study (median time 113 days, 95 % CI: 85–141 days). This difference was statistically significant based on non-overlapping 95 % CIs suggesting faster and greater benefit to patients from the BIW dosing regimen than QW dosing regimen.

Analysis of HRQoL measures demonstrated statistically significant (*p* < 0.01) improvement of scores from baseline in response to etanercept treatment for all parameters in both treatment groups at both 12 weeks and 24 weeks. Furthermore, the differences in the improvements observed for DLQI and EQ-5D scores at week 12 between the BIW and QW treatment groups were statistically significant (*p* < 0.05). These data parallel the data observed for the efficacy analyses for this subset of patients.

## Conclusion

The subset analysis reported here demonstrates that patients with moderate-to-severe plaque psoriasis from six countries in Asia, Central Europe and Latin America respond to etanercept treatment in a manner similar to that observed in patients from the United States and Western Europe. In all analyses, compared with QW treatment, BIW treatment appears to be more beneficial with more rapid and greater response. The results for this Asian, Central European and Latin American subpopulation of the PRISTINE trial, as well as the overall study population, showed etanercept was well tolerated by patients at both the BIW and QW dosages and there were no differences in safety parameters between the two treatment groups [8]. In conclusion, this analysis suggests that the guidelines for the treatment of psoriasis in the United States and Europe can be applied to populations from other parts of the world as well.

## Abbreviations

BIW: Twice weekly
BSA: Body surface area
CI: Confidence interval
DLQI: Dermatology life quality index
EQ-5D: EuroQoL–5 dimension
FACIT: Functional activity in chronic therapy
HRQoL: Health-related quality of life
PASI: Psoriasis area and severity index
PGA: Physician global assessment of psoriasis
QW: Once weekly
WPAI: Work productivity and activity impairment
